# Association of the *CD226* (*DNAM-1*) Gly307Ser polymorphism with juvenile idiopathic arthritis

**DOI:** 10.1186/1546-0096-9-S1-P286

**Published:** 2011-09-14

**Authors:** THCM Reinards, HM Albers, DMC Brinkman, SSM Kamphuis, MAJ van Rossum, EPAH Hoppenreijs, HJ Girschick, C Wouters, RK Saurenmann, JJ Houwing-Duistermaat, REM Toes, TWJ Huizinga, R ten Cate, MW Schilham

**Affiliations:** 1 Leiden University Medical Center, Leiden, The Netherlands; 2 Erasmus Medical Center, Rotterdam, The Netherlands; 3 Academic Medical Center, Amsterdam, The Netherlands; 4 Radboud University Medical Center, Nijmegen, The Netherlands; 5 University of Würzburg, Würzburg, Germany; 6 University Hospital Gasthuisberg, Leuven, Belgium; 7 University Children's Hospital, Zürich, Switzerland

## Background

Recent genetic studies have reported an association of the Gly307Ser single nucleotide polymorphism (SNP) in the CD226 gene with susceptibility to multiple autoimmune diseases. *CD226* or *DNAM-1* is a type 1 membrane protein belonging to the Ig-superfamily and is involved in the adhesion and co-stimulation of T cells and NK cells. A trend towards association of this SNP with Juvenile Idiopathic Arthritis (JIA) was recently reported, but was not statistically significant (p=0.13) (Genes Immun. 2010 Mar;11(2):194-8).

## Aim

We performed a case-control genetic association study to investigate whether *CD226* Gly307Ser is associated with susceptibility to JIA.

## Methods

*CD226* Gly307Ser was genotyped in 667 JIA cases and 1320 healthy controls, both of North-West-European white origin. Allele frequencies were compared. Patients with oligoarticular (persistent and extended), polyarticular (rheumatoid factor negative and positive) and systemic JIA have been included and were analyzed separately as well as grouped together. A meta-analysis of our study combined with the previously published study was performed.

## Results

*CD226* Gly307Ser was significantly associated with susceptibility to JIA (p=0.002, OR=1.23, 95% CI: 1.08-1.41), particularly in the persistent oligoarticular subtype (p=0.0008, OR=1.38, 95% CI: 1.14-1.67). The meta-analysis (of all subtypes grouped together) confirmed the association with JIA (p=0.003, OR=1.14, 95% CI: 1.05-1.23).

## Conclusion

This study provides evidence for a novel JIA susceptibility locus. Additionally, a subtype-specific association of *CD226* with persistent oligoarticular JIA has been identified. This finding is in line with the hypothesis that this oligoarticular subtype is not only phenotypically, but also genetically different from the polyarticular subtypes, including extended oligoarthritis.

